# Validity and Psychometric Evaluation of the Chinese Version of the 5-Item WHO Well-Being Index

**DOI:** 10.3389/fpubh.2022.872436

**Published:** 2022-03-30

**Authors:** Sai-fu Fung, Chris Yiu Wah Kong, Yi-man Liu, Qian Huang, Zike Xiong, Zhiquan Jiang, Fangfang Zhu, Zhenting Chen, Kun Sun, Huiqin Zhao, Ping Yu

**Affiliations:** ^1^Department of Social and Behavioural Sciences, City University of Hong Kong, Kowloon, Hong Kong SAR, China; ^2^School of Economics, Finance and Marketing, Royal Melbourne Institute of Technology, Melbourne, VIC, Australia; ^3^University Administrative Office, Guangzhou Huashang College, Guangzhou, China; ^4^Department of Sports Training, Xi'an Physical Education University, Xi'an, China; ^5^Society Hub, The Hong Kong University of Science and Technology (Guangzhou), Guangzhou, China; ^6^School of Economics and Trade, Guangzhou Huashang College, Guangzhou, China; ^7^School of Data Sciences, Guangzhou Huashang College, Guangzhou, China; ^8^Managing Director Office, Global Business College of Australia, Melbourne, VIC, Australia; ^9^General Manager Office, Edvantage Institute Australia, Melbourne, VIC, Australia

**Keywords:** wellbeing, WHO-5, CFA, Chinese, validation, student

## Abstract

**Aims:**

This article evaluates the psychometric properties of the Chinese version of the 5-item WHO Well-Being Index (WHO-5) in mainland China.

**Methods:**

Two cross-sectional studies with 1,414 participants from a university in China were conducted. The Chinese version of the WHO-5 was assessed to determine its internal consistency, concurrent validity, factorial validity, and construct validity.

**Results:**

The results indicate that the WHO-5 is unidimensional and has good internal consistency, with Cronbach's *a* = 0.85 and 0.81 in Study 1 (*n* = 903) and Study 2 (*n* = 511), respectively. The findings also demonstrate that the WHO-5 has good concurrent validity with other well-established measures of wellbeing, self-efficacy, self-esteem, and mental wellbeing. The results of confirmatory factor analysis also suggest that the scale has a good model fit.

**Conclusions:**

This study provides empirical data demonstrating that the Chinese version of the WHO-5 has good psychometric properties. The scale can be a useful measure in epistemological studies and clinical research related to wellbeing in Chinese populations.

## Introduction

The WHO 5-item Well-Being Index (WHO-5) is a well-known psychological measurement scale that assesses subjective wellbeing through a non-symptomatic and positively worded self-report instrument for a 14-day period (1, 2). The development of the scale began with its longer versions, the WHO-28 and WHO-10 (3–5). By 1998, researchers had successful reduced the instrument to a more user-friendly 5-item scale using a 6-point Likert scale, ranging from 0 (*at no time*) to 5 (*all of the time*) (6). Since then, it has gained worldwide popularity as a screening tool in epidemiological research on areas such as depression, suicidal ideation, infertility, and diabetes (7–9). Recently, numerous studies have applied the WHO-5 to measure comprehensive bio-psychosocial wellbeing (10, 11), indicating an attempt at wider application.

The wider application of the scale depends on its continuous improvement in work by scholars and clinical researchers translating and validating its applicability in Western, Asian, and Latin American countries (6, 11–13). However, in its positive application in various cultures, the construct validity of the WHO-5 has been overlooked (14, 15), with researchers focusing on exploratory factor analysis (EFA) to evaluate the unidimensional latent construct of the scale (16). As such, there are various validation studies on WHO-5 only evaluated the factorial validity of the measure with EFA (9, 17). EFA cannot constrain data, whilst confirmatory factor analysis (CFA) imposes meaningful constraints in assessing the validity of a measure (15). The development and use of CFA was a crucial step in scale validation (18). Yet, surprisingly, WHO-5 assessments using CFA are scarce (1). To the best of our knowledge, this is the first validation study on the Chinese version of WHO-5 with empirical data from two cross-sectional studies using both EFA and CFA to evaluate its construct validity.

This study aimed to fill this gap by conducted two studies. One study evaluated the Chinese version of the WHO-5 with Chinese university students to reveal its psychometric properties. The second study was aimed at validating and confirming the factors in the WHO-5 to reveal its robustness in CFA. Last, the concurrent validity of the WHO-5 with several well-established construct-related concepts related to mental wellbeing (6, 8, 9, 12, 19), life satisfaction, self-esteem, and self-efficacy (1, 20, 21) was also investigated.

Overall, this study provides empirical evidence of the psychometric properties of the Chinese version of the WHO-5, as well as evidence confirming its academic development and application. The validation should be beneficial for comprehensive psychological measurements of other student populations in China. The wider application of this validated scale should help practitioners monitor the mental health and wellbeing of Chinese university students.

## Materials and Methods

### Participants

To evaluate the psychometric properties of the WHO-5, two cross-sectional studies were conducted in a university in Guangdong, China with 1,414 valid participants. We have set 95% confidence level and 5% margin of error when determining the sampling size. The minimum sample size was 377 in the research setting (22). Study 1 took place between June and July 2018 with 903 undergraduate students with an average age of 20.56 years (SD = 2.75 years) who voluntarily participated. The sample comprised 111 male and 792 female participants. In addition, 511 students participated in Study 2 from April to May 2019. The margin of error for the above samples was 3.12% (*n* = 903) in Study 1 and 4.19% (*n* = 511) in Study 2. The sample comprised 85.5% female and 14.5% male participants with an average age of 20.41 years (SD = 2.49 years). The gender ratio reflected the overall student demographic profile of the setting.

Both studies used the university's student intranet system to recruit participants and distribute the questionnaire. The collected data stored on the system were completely anonymous. The participants were invited to participate on a voluntary basis. Informed consented was obtained from all of the participants. Parental consent was not required as the participants are all over 18 years old. The participants were allowed to withdraw at any time during the data collection process. The studies were approved by the university's research ethics committee. The entire research process strictly adhered to relevant national and international ethical standards.

### Measures

The WHO-5 consists of five items with a 6-point Likert-type scale ranging from 0 (*at no time*) to 5 (*all of the time*) that measure wellbeing. A higher score indicates a higher level of wellbeing (5, 16, 23). The development of the Chinese WHO-5 used standard translation and back-translation procedures by two translators with proficiency in both English and Simplified Chinese (24). To avoid geographical and cross-cultural differences within China, two pilot studies were conducted in Xi'an, Shaanxi and in Guangzhou, Guangdong with 10 pilot participants with at least a degree qualification (25, 26). None of the participants reported any difficulty understanding the questions. The data collected from the pilot studies were excluded from subsequent analysis.

The Satisfaction with Life Scale (SWLS) is made up of five items with a 7-point Likert-type scale, ranging from 1 (*strongly disagree*) to 7 (*strongly agree*) (27–30). The Chinese version of the SWLS was validated by Bai et al. (31) with a nationally representative sample. The Cronbach's alpha in Study 1 and Study 2 are 0.883 and 0.819, respectively.

The Personal Well-Being Index (PWI) is evaluated on an 11-point Likert-type scale (0 = *no satisfaction at all* to 10 = *completely satisfied*) with seven questions related to various quality of life domains, including standard of living, health, achieving in life, relationships, safety, community-connectedness, and future security (*a* = 0.902 in Study 1; 0.916 in Study 2). The original scale developer validated the Chinese version (32). The Cronbach's alpha of PWI in both Study 1 and Study 2 are above the acceptable range with 0.902 and 0.916.

The Rosenberg Self-Esteem (RSE) Scale comprises 10 statements (with five items reverse-coded) evaluated using a 4-point Likert-type scale (1 = *strongly disagree* to 4 = *strongly agree*) (33, 34). Wu et al. (34) validated the Chinese version of the RSE with 982 adolescents. The current study also reported the acceptable alpha coefficient (Study 1 = 0.830; Study 2 = 0.755).

The General Self-Efficacy Scale (GSE) consists of 10 items on a 4-point Likert-type scale (1 = *not at all true* to 4 = *exactly true*) (35–37). The Chinese version of the GSE has recently been validated (34, 38). The GSE in Study 1 and Study 2 with Cronbach's alpha 0.903 and 0.884, respectively.

The Short Warwick Edinburgh Mental Well-Being Scale (SWEMWBS) evaluates hedonic and eudaimonic wellbeing with a 5-point scale (1 = *none of the time* to 5 = *all of the time*) with seven positively worded questions (14, 39). The Chinese version has been validated in both school and clinical settings (40–43). The Cronbach's alpha in Study 1 = 0.884 and Study 2 = 0.824.

Last, the 12-item General Health Questionnaire (GHQ-12) contains 12 items to evaluate the severity of health-related problems with a 4-point scale (44). Higher scores indicate worse health. The Chinese version has been validated in various contexts (45, 46). The Cronbach's alpha in Study 1 and Study 2 are 0.773 and 0.751, respectively.

### Ethical Statement

This study was conducted in accordance with the ethical standards of City University of Hong Kong and Guangzhou Huashang College research ethics committee and with the 1964 Helsinki declaration and its later amendments. Informed consent was obtained from all individual participants included in the study.

### Procedure

Using data from Study 1 (*n* = 903) and Study 2 (*n* = 511), the internal consistency of the WHO-5 was evaluated using Cronbach's alpha (47) and McDonald's omega (48–50), and the corrected item-total correlations between the five items were examined (51, 52).

EFA with principal component analysis was used to evaluate the factorial validity of the WHO-5 (1, 18, 53). To avoid the potential problem of overfitting when conducting EFA and CFA on the same dataset (54), EFA was only conducted on the sample from Study 2 (*n* = 511). EFA adopted the cut-off values of the Kaiser–Mayer–Olkin (KMO) test (>0.70) and Bartlett's test of sphericity (*p* < 0.01). In addition, the identified factors should have eigenvalues >1 and their loadings should be >0.350 (51, 55).

The construct validity of the WHO-5 was further evaluated with CFA based on the sample obtained from Study 1 (*n* = 903) (56). Recent studies on CFA have suggested that the maximum likelihood estimator is inappropriate for a scale measured with ordinal items (57); hence, a diagonally weighted least squares (DWLS) estimator was used (58–60) in Model 1 and 2. The recent simulate study recommended that maximum likelihood with mean- and variance-adjusted likelihood ratio test (MLMV) yields better results. Hence, we adopted this estimator in Model 3 (61, 62). The following well-established fit indices were used to evaluate the model fit: comparative fit index (CFI) > 0.90, Tucker–Lewis index (TLI) > 0.90, root mean square error of approximation (RMSEA) < 0.08, and root mean square residual (SRMR) < 0.08 (51, 63–65). In addition, the ratio of the chi-square test statistic to degrees of freedom, χ^2^/df ≤ 3, was used to determine an acceptable model fit (66–69) with the exception of Model 3, as the chi-square value of MLMV cannot be used for regular way (70).

Concurrent validity was assessed using the data from both Study 1 (*n* = 903) and Study 2 (*n* = 511) along with other validation constructs or measures reported in relevant studies on the WHO-5 (18, 71). Specifically, the WHO-5 has been shown to be significantly positively correlated with life satisfaction, self-esteem, and self-efficacy (1, 20, 21) and negatively correlated with mental health and psychiatric morbidity (6, 8, 12, 19). Hence, the following scales were used to evaluate the concurrent validity of the WHO-5: SWLS, PWI, RSE, GSE, SWEMWBS, and GHQ-12.

The above analyses were conducted using the R (3.6.3) computing environment with the lavaan package 0.6-5 (72), Mplus 8.5 (70), and IBM SPSS 26.0.

## Results

### Internal Consistency

Table 1 presents the descriptive statistics, including the mean, standard deviation, skewness, kurtosis, corrected item-total correlations, and Cronbach's alpha (if an item was deleted) for the five items of the WHO-5, based on the data from Study 1 (*n* = 903) and Study 2 (*n* = 511). The results showed that the WHO-5 had good internal consistency. The corrected item-total correlations for the WHO-5 ranged from 0.585 to 0.751 in Study 1 and from 0.529 to 0.618 in Study 2. The Cronbach's alpha and McDonald's omega values were above the acceptable range, with *a* = 0.85 and ω = 0.86 in Study 1 and *a* = 0.81 and ω = 0.82 in Study 2. There were no significant differences, and relationships were observed in the scale scores by gender, based on the independent-sample *t*-test and correlation results.

**Table 1:** Descriptive statistics for the WHO-5 items in Study 1 and Study 2.

**Item**	** $\bar{x}$ **	**SD**	**sk**	**ku**	**r*_***it***_***	***a_***iid***_***
**Study 1**						
WHO5-1	3.80	0.993	−0.242	0.025	0.669	0.819
WHO5-2	3.54	0.992	0.082	0.019	0.709	0.808
WHO5-3	3.56	0.935	0.136	0.096	0.751	0.799
WHO5-4	3.37	1.056	0.175	−0.105	0.585	0.843
WHO5-5	3.47	0.933	0.145	0.067	0.611	0.834
**Study 2**						
WHO5-1	3.64	0.968	−0.405	0.341	0.588	0.780
WHO5-2	3.53	0.924	−0.188	0.294	0.618	0.771
WHO5-3	3.52	0.957	−0.099	−0.080	0.683	0.751
WHO5-4	3.38	1.070	−0.152	−0.166	0.529	0.801
WHO5-5	3.36	0.958	0.055	−0.184	0.598	0.777

*R, Reversed item; sk, Skewness; ku, Kurtosis; r_it_, Corrected item-total correlations; a_iid_, Cronbach's alpha, if item deleted*.

### Factorial Validity

Table 2 illustrates the EFA results using principal component analysis for Study 2 (*n* = 511). The results of the KMO and Bartlett's test of sphericity for the WHO-5 were 0.804 (χ^2^ = 833.749, *p* < 0.001), indicating appropriate scale construction. The scale was unidimensional with only one factor with an eigenvalue >1. The factor loadings ranged from 0.478 to 0.674, explaining 57.593% of the total variance.

**Table 2:** Exploratory factor analysis with principal component analysis on WHO-5 items.

**Item**	**Study 2**
1. I have felt cheerful and in good spirits.	0.569
2. I have felt calm and relaxed.	0.598
3. I have felt active and vigorous.	0.674
4. I woke up feeling fresh and rested.	0.478
5. My daily life has been filled with things that interest me.	0.561

### Construct Validity

**Figure 1:**
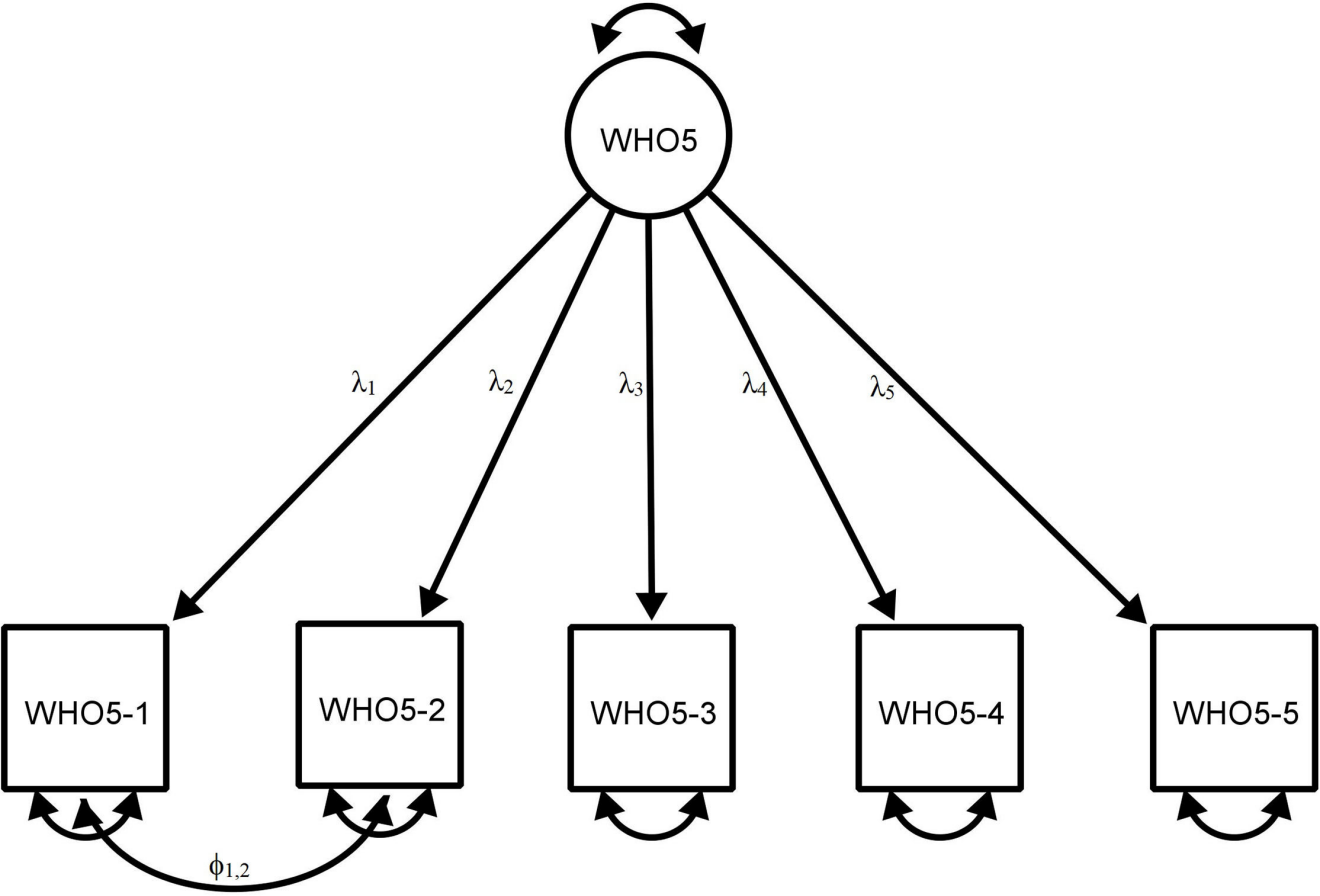
Estimated model of the 5-item WHO Well-Being Index.

Table 3 and Figure 1 show the CFA results for the WHO-5 based on Study 1 (*n* = 903). Model 1 evaluated the WHO-5 based on a single factor, without correlating the error terms. The results generally satisfied the criteria for an adequate model fit, with CFI = 0.996, TLI = 0.992, and SRMR = 0.037. However, the following two indices failed to fit the model: χ^2^ (50.536)/5 = 10.107 and RMSEA = 0.100. Following recent studies on the WHO-5 (73), Model 2 re-evaluated the scale, with the error correlations based on the modification indices. It included one covariance factor between the error terms for the WHO5-1 and WHO5-2. The CFA results indicated a good fit of the model, with χ^2^ (10.988)/4 = 2.747, *p* < 0.05, SRMR = 0.019, CFI = 0.999, TLI = 0.998, and RMSEA = 0.044. Model 3 further evaluated the WHO-5 with MLMV estimator without correlated errors. The results indicated that the WHO-5 generally had an adequate fit with a unidimensional factor structure without any *post-hoc* modifications, with SRMR = 0.030, CFI = 0.974, TLI = 0.947, and RMSEA = 0.080 (Model 3).

**Table 3:** Factor loadings and fit indices in confirmatory factor analysis for the WHO-5 (see Figure 1 for estimated model).

		**Study 1**		
**Item**		**Model 1**	**Model 2**	**Model 3**
WHO5-1	λ_1_	0.799	0.725	0.756
WHO5-2	λ_2_	0.838	0.769	0.798
WHO5-3	λ_3_	0.856	0.905	0.829
WHO5-4	λ_4_	0.662	0.676	0.632
WHO5-5	λ_5_	0.693	0.708	0.654
**Residual correlations**
WHO5-1−WHO5-2	ϕ_1, 2_	-	0.160	-
**Model fit**
*N*		903	903	903
RMSEA		0.100	0.044	0.080
RMSEA 90% CI		0.076–0.127	0.014–0.076	0.064–0.115
SRMR		0.037	0.019	0.030
χ^2^		50.536	10.988	-
df		5	4	-
χ^2^/df		10.107	2.747	-
CFI		0.996	0.999	0.974
TLI		0.992	0.998	0.947

### Concurrent Validity

The results of Study 1 (*n* = 903) replicated the relationships between the WHO-5 and the other construct-related scales suggested in the wellbeing literature (Table 4). In particular, the WHO-5 had significant and strong positive relationships with the SWLS (*r* = 0.507, *p* < 0.001) and PWI (*r* = 0.500, *p* < 0.001). The RSE (*r* = 0.351, *p* < 0.001) and GSE (*r* = 0.394, *p* < 0.001) also had a moderate positive relationship with the WHO-5. In general, these results were similar in Study 2 (*n* = 511).

**Table 4:** Correlations between the WHO-5 in relation to other well-established scales.

**Scale**	**Study 1** **WHO-5**	**Study 2** **WHO-5**
Satisfaction with Life Scale (SWLS)	0.507	0.519
Personal Well-Being Index (PWI)	0.500	0.499
Rosenberg self-esteem (RSE) scale	0.351	0.478
General self-efficacy scale (GSE)	0.394	0.408
Short Warwick Edinburgh Mental Well-being Scale (SWEMWBS)	0.438	0.537
12-item General Health Questionnaire (GHQ-12)	−0.342	−0.411

*All correlations are significant at the 0.001 level (2-tailed)*.

Regarding the concurrent validity of the WHO-5, the scale was expected to demonstrate a negative relationship with the psychological symptom-related scales. As predicted, the WHO-5 was positively related to the SWEMWBS, a scale in which a lower score indicates psychiatric morbidity, with *r* = 0.438 (*p* < 0.001) in Study 1 and *r* = 0.537 (*p* < 0.001) in Study 2 (*n* = 511). The results also demonstrated that the Chinese version of the WHO-5 had a significant moderate negative relationship with the GHQ-12 in Study 1 (*r* = −0.342, *p* < 0.001) and Study 2 (*r* = −0.411, *p* < 0.001). In summary, the WHO-5 showed good concurrent validity based on Pearson's correlation coefficients.

## Discussion

Subjective wellbeing is an important denominator in various mental health issues. The WHO-5 offers a set list for evaluating the effectiveness of treatment with a friendly, easy to understand, and non-invasive assessment. Its wider application to assess psychological responses to various types of disease is apparent in its capacity for early and effective identification. By validating the Chinese version of the WHO-5, this study opens its wider application to investigate the wellbeing of Chinese undergraduate students, such as stress-related issues in work and education settings (2). Specifically, the results of this study showed that the Chinese version of the WHO-5 has good psychometric properties. Indeed, the results indicated that the scale has good internal consistency, with Cronbach's alpha values of 0.85 and 0.81 in Study 1 and Study 2, respectively, similar to the values reported in recent WHO-5 studies (ranging from 0.78 to 0.85) based on adolescents and adults in various settings (8, 11, 12, 19, 73). The unidimensional factor structure of the Chinese version of the WHO-5 replicated that of the original WHO-5 (5, 16, 23). The results in this study also showed that the WHO-5 has good concurrent validity with well-established measures related to wellbeing, self-esteem, self-efficacy, and mental wellbeing. In short, the Chinese version of the WHO-5 is suitable for studying the wellbeing of Chinese university students.

This study contributes to the measurement of wellbeing in the following ways. First, this study is one of the first to validate the Chinese version of the WHO-5 for the student population. Although many epistemological studies have used the WHO-5 in Chinese contexts (74–80), there is a paucity of studies validating the Chinese version of the scale. In addition, most of the WHO-5 studies conducted in other countries have focused on clinical populations (1, 9, 12). As such, many existing studies reported that the WHO-5 has been used as outcome measure for the clinical trials amongst the patients with medical conditions related to oncology, endocrinology, otolaryngology, etc. (2). The findings of this study indicated that the WHO-5 is a reliable tool to address mental health challenges in a non-clinical sample, which can contribute to the field of public health.

The second contribution of this study is to provide empirical data to evaluate the construct validity of the WHO-5 through CFA. Validation studies have mainly evaluated construct validity using only EFA (4, 6, 12, 19). However, validation scholars have advocated the use of CFA (18, 38, 81). Many recent studies have demonstrated that scales developed and validated using only EFA may suffer from various methodological issues, such as poor factorial validity and difficulty replicating the factor structure (82, 83). This study conducted two cross-sectional studies to evaluate the scale through both EFA (Study 2) and CFA (Study 1) to avoid the above issues.

This study may have the following limitations. First, the results of this study were based on two cross-sectional studies conducted in a Chinese university located in Guangdong Province in southern China. This may limit the generalizability of the findings to Chinese society or to the Chinese diaspora as a whole. Second, the construct-related measures used in this study are limited by the availability of validated Chinese versions of the scales related to wellbeing, self-efficacy, self-esteem, and mental wellbeing, which may be slightly different from the measures used by the original developers. To overcome this potential limitation, we adopted measures and concepts that have been frequently discussed and applied in WHO-5 studies (1, 6, 8, 9, 12, 19–21). The last potential limitation is related to the *post-hoc* modifications in CFA to meet all of the criteria for a good model fit. Model 1 (Table 3) reported that SRMR, CFI, and TLI met the criteria for a good model fit and that χ^2^/df and RMSEA did not. We are fully aware of the discussion about avoiding the use of correlated error terms in CFA without strong justifications (84, 85). Recent WHO-5 validation studies that used CFA have also correlated the error terms (8, 15, 73). This practice has been justified in the literature (86–90). Hence, after correlating the error terms for items 1 and 2, Model 2 showed that the WHO-5 met all of the stringent indices for a good model fit [χ^2^ (10.988)/4 = 2.747, *p* < 0.05, SRMR = 0.019, CFI = 0.999, TLI = 0.998, RMSEA = 0.044], indicating that the Chinese version has good construct validity. To overcome this limitation, we computed additional CFA analysis with MLMV estimator in Model 3 without correlating any error terms between the items. The results fulfilled the requirement of adequate model fit, with SRMR = 0.030, CFI = 0.974, TLI = 0.947, and RMSEA = 0.080 (Table 3).

Future studies should include wider population samples, such as young working adults, and non-university youth populations, such as primary and secondary Chinese students. By establishing the broader applicability of the WHO-5 to social work and counseling interventions, the rapid screening enabled by this instrument will provide a viable means of detecting the emotional and psychological wellbeing of young people, making early intervention possible, especially for stress-related issues at work or school. If longitudinal research were conducted, the scale would be available to examine the psychosocial wellbeing of Chinese primary and secondary students. The important data obtained would provide teachers, parents, and students themselves with insight into their psychosocial and emotional health. Another direction could be to compare the subjective wellbeing of primary, secondary, university, and working youth populations at these important stages of development.

## Conclusions

In summary, this study validated the Chinese version of the WHO-5. The findings indicate that the scale has good internal consistency, concurrent validity, factorial validity, and construct validity. The results suggest that the Chinese version of the WHO-5 is a valid measure of the mental wellbeing of Chinese university students. The findings may encourage researchers and practitioners to use this scale in epidemiological research. However, additional work is needed to confirm the psychometric properties of the WHO-5 with more generalizable samples in other contexts.

## Data Availability Statement

The raw data supporting the conclusions of this article will be made available by the authors, without undue reservation.

## Ethics Statement

The studies involving human participants were reviewed and approved by Research Ethics Committee of the Guangzhou Huashang College. The patients/participants provided their written informed consent to participate in this study.

## Author Contributions

S-fF has worked in the conception and design of the study. Y-mL, QH, ZX, ZJ, FZ, ZC, KS, HZ, and PY have worked on data collection. S-fF and CK have performed statistical data analyses, interpretation, and have written the article. All authors have critically reviewed the manuscript and approved its last version for publication.

## Conflict of Interest

The authors declare that the research was conducted in the absence of any commercial or financial relationships that could be construed as a potential conflict of interest.

## Publisher's Note

All claims expressed in this article are solely those of the authors and do not necessarily represent those of their affiliated organizations, or those of the publisher, the editors and the reviewers. Any product that may be evaluated in this article, or claim that may be made by its manufacturer, is not guaranteed or endorsed by the publisher.
